# Pathogen‐induced expression of a blight tolerance transgene in American chestnut

**DOI:** 10.1111/mpp.13165

**Published:** 2021-11-28

**Authors:** Erik Carlson, Kristen Stewart, Kathleen Baier, Linda McGuigan, Tobi Culpepper, William Powell

**Affiliations:** ^1^ Department of Environmental Biology SUNY College of Environmental Science and Forestry Syracuse New York USA

**Keywords:** blight tolerance, *Castanea dentata*, *Cryphonectria parasitica*, oxalate oxidase (*OxO*), *win3.12* poplar promoter

## Abstract

American chestnut (*Castanea dentata*) is a susceptible host of the invasive necrotrophic fungus *Cryphonectria parasitica*, which causes chestnut blight disease. The fungal pathogen attacks chestnut stems by invading wounded tissue and secreting oxalate. This process leads to the death of infected host cells and the formation of cankers, eventually girdling stems and killing the tree above the infections. To reduce damage caused by fungal oxalate, American chestnut has been genetically engineered to express a wheat oxalate oxidase (*OxO*). This enzyme degrades the oxalate produced by the pathogen and confers elevated tolerance to *Cryphonectria parasitica* infection. We report new lines of transgenic American chestnut that have been developed with the *win3.12* inducible promoter from poplar (*Populus deltoides*) driving *OxO* expression. This promoter is responsive to both wounding and pathogen infection, with a low level of baseline expression. Targeted expression of *OxO* to wounded and infected tissue is sought as an alternative to constitutive expression for potential metabolic resource conservation and transgene stability over the long lifetime of a tree and over successive generations of breeding. Transgenic *Castanea dentata* lines harbouring the *win3.12‐OxO* construct were evaluated for transgene expression patterns and tolerance to chestnut blight infection. *OxO* transcript levels were low in uninfected plants, but robust infection‐induced expression levels were observed, with one transgenic line reaching levels comparable to those of previously characterized CaMV35S*‐OxO* lines. In chestnut blight infection bioassays, *win3.12‐OxO* lines showed elevated disease tolerance similar to blight‐resistant Chinese chestnut (*Castanea mollissima*) controls.

## Graphical abstract

In transgenic *Castanea dentata*, a promoter from *Populus deltoides* is strongly induced by *Cryphonectria parasitica* infection and effectively drives expression of the chestnut blight tolerance gene oxalate oxidase (*OxO*).

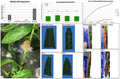

## 1 INTRODUCTION

The American chestnut (*Castanea dentata*) was once an ecologically, economically, and culturally important tree within its range in the eastern forests of North America (Diamond et al., 2000). It is now considered functionally extinct due to chestnut blight (Westbrook et al., 2019) caused by *Cryphonectria parasitica*, a non‐native fungal pathogen. The tree's susceptibility is in large part due to the inability of *C. dentata* to detoxify the oxalate (oxalic acid) secreted by the pathogen (Havir & Anagnostakis, 1983; Lovat & Donnelly, 2019). Asian chestnut species such as Chinese chestnut (*Castanea mollissima*) appear to have enhanced oxalic acid tolerance compared to American chestnut (Newhouse, Polin‐McGuigan, et al., 2014), which is hypothesized to be the product of a metabolic pathway controlled by multiple genes including oxalyl‐CoA synthetase as previously observed in *Arabidopsis thaliana* (Foster et al., 2012). The heterologous expression of a wheat (*Triticum aestivum*) oxalate oxidase (*OxO*) in *C. dentata* has been demonstrated to confer elevated tolerance to infection by chestnut blight (Newhouse, Polin‐McGuigan, et al., 2014; Zhang et al., 2013). Tolerance is a form of disease resistance whereby a plant host is able to survive pathogen infection by limiting damage without killing the pathogen itself (Pagán & García‐Arenal, 2018). In the case of chestnut blight, the terms blight “tolerance” and “resistance” are often used interchangeably, although blight tolerance has recently become the preferred descriptor (Powell et al., 2019; Steiner et al., 2017; Westbrook et al., 2019; Zhang et al., 2013). Instead of directly attacking the pathogen, *OxO* works by degrading oxalic acid into hydrogen peroxide and carbon dioxide, two products that are not harmful to American chestnut. Hydrogen peroxide is a compound commonly used in plant signalling responses to infection; its production within blight‐infected tissue could potentially signal innate defences within American chestnut (Barakat et al., 2012). Such defences might include the formation of a lignified zone and a wound periderm for infection containment (Hebard et al., 1984; Lovat & Donnelly, 2019).

Two different promoters have controlled the expression of *OxO* in previously characterized lines of transgenic American chestnut: the soybean (*Glycine max*) vascular‐specific promoter *VspB* and the CaMV 35S constitutive promoter (Newhouse, Polin‐McGuigan, et al., 2014; Zhang et al., 2013). Some *VspB‐OxO* events such as Darling 4 showed statistically significant increases in tolerance to chestnut blight when compared to wild‐type American chestnut, but tolerance did not reach levels consistent with resistant Chinese chestnut controls (Newhouse, Polin‐McGuigan, et al., 2014; Zhang et al., 2013). To further enhance blight tolerance, higher levels of *OxO* expression were sought. Gene constructs using CaMV 35S driving *OxO* expression were generated and used to transform American chestnut. These transgenic events had higher levels of *OxO* expression than previous *VspB‐OxO* events, resulting in higher levels of chestnut blight tolerance similar to those of resistant Chinese chestnut controls (Zhang et al., 2013). The expression levels of *OxO* in resistant transgenic lines were determined and a theoretical threshold level of expression necessary for enhanced blight tolerance was established (Zhang et al., 2013). These blight‐tolerant transgenic lines have been intensively studied for potential environmental interactions (Brown et al., 2019; D'Amico et al., 2015; Goldspiel et al., 2018; Newhouse et al., 2018; Steiner et al., 2017) and at the time of publication for this study are undergoing US regulatory review for release and potential use in restoration.

The *win3.12* promoter, originally isolated from poplar, was shown in previous research to be inducible by wounding and pathogen infection in transgenic plants (Hollick & Gordon, 1993; Liang et al., 2002; Yevtushenko & Misra, 2007, 2019; Yevtushenko et al., 2004). This promoter originates from a Kunitz‐protease inhibitor gene from a *Populus trichocarpa* × *P*. *deltoides* hybrid (later determined to be inherited from the *P*. *deltoides* parent) that expresses in response to wounding (Bradshaw et al., 1990, 1991; Hollick & Gordon, 1993, 1995; Parsons et al., 1989). Genetic analysis of *win3.12* sequences revealed key elements in its promoter, including five clustered W‐box motifs with a TGAC core sequence that serve as binding sites for pathogen‐associated WRKY transcription factors (Yevtushenko et al., 2005). WRKY transcription factors are a large family of proteins that activate expression of genes containing corresponding response elements (Yu et al., 2001). The promoter also contains a wound‐induced delayed expression element (WIDE) that is associated with tissue repair gene pathways that are activated by wounding (Hernandez‐Garcia & Finer, 2016). The WIDE element contained in the promoter is induced immediately in response to wounding, but also increased expression up to several days later (Hernandez‐Garcia & Finer, 2016). Pathogen‐induced transcription elicits a much stronger response than induction caused by wounding (Yevtushenko et al., 2004) and the promoter is responsive to a broad spectrum of plant diseases (Yevtushenko & Misra, 2007).

The *win3.12* inducible promoter has low baseline levels of expression, but expresses at high levels in response to wounding and infection. Blight infections typically initiate at wound sites (Hebard et al., 1984; Lovat & Donnelly, 2019); a promoter that targets defence transgene expression to wounds may limit damage in the crucial early stages of infection. Pathogen induction of *win3.12* is a separate process from wound induction, which would prevent the maximum level of expression in uninfected wound tissue (Hernandez‐Garcia & Finer, 2016; Yevtushenko et al., 2004). This two‐step expression response serves to maximize expression where it is needed while at the same time limiting expression when it is not. American chestnut is a long‐lived tree species; the potential energetic savings of an inducible promoter compared to a constitutive promoter over a tree's lifetime could be significant.

The *win3.12* promoter was combined with the blight tolerance gene *OxO* and introduced into American chestnut via *Agrobacterium*‐mediated transformation. The objective of this study was to characterize the expression patterns of the *win3.12* promoter in transgenic *C. dentata*; a secondary goal was to determine the effectiveness of the *win3.12* promoter in driving *OxO* expression for the enhancement of blight tolerance. It was hypothesized that the *win3.12‐OxO* transgene would be induced by blight infection and result in enhanced disease tolerance in American chestnut.

## 2 RESULTS

### 2.1 Transformation of American chestnut with *win3.12‐OxO* and screening of transgenic events

**FIGURE 1:**
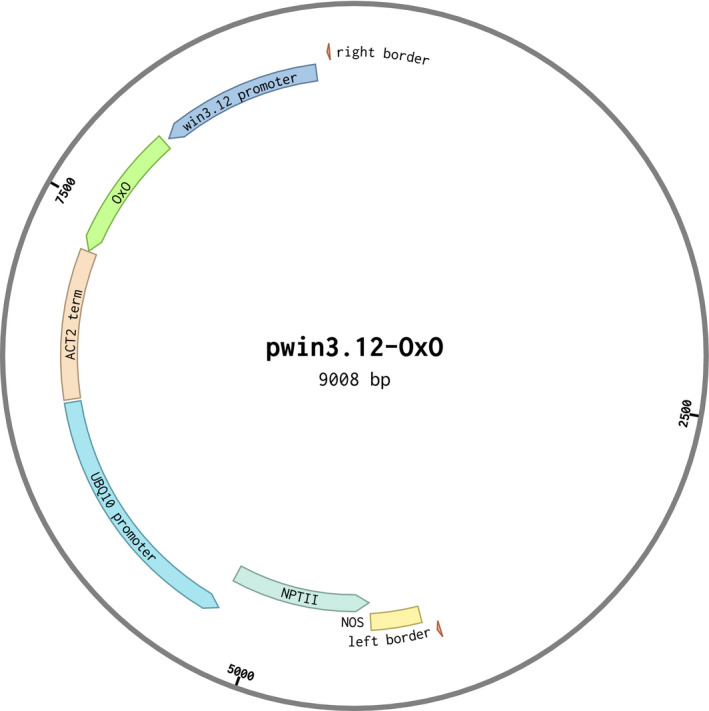
Sequence map of pwin3.12‐OxO. The 823 bp *win3.12* promoter from poplar was inserted upstream of the 675 bp *Triticum aestivum* oxalate oxidase (*OxO*) coding sequence and the 473 bp *ACT2* terminator from *Arabidopsis*. The neomycin phosphotransferase II (*NPTII*) antibiotic resistance gene under the control of *Arabidopsis UBQ10* promoter was used for the selection of embryo transformants

The *win3.12‐OxO* vector (Figure 1) was constructed with the *win3.12* promoter from poplar and the oxalate oxidase (*OxO*) gene from wheat and transformed into American chestnut somatic embryos (McGuigan et al., 2020). Twenty‐two independent transgenic embryo lines were recovered and tested for insert copy number using quantitative PCR (qPCR). Transgenic embryo lines contained 1–7 copies of *win3.12‐OxO*. All high‐copy number lines (i.e., ≥3 copies) were excluded due to the reduced predictability of inheritance patterns in subsequent breeding generations (Newhouse, Polin‐McGuigan, et al., 2014) and to reduce the risk of gene silencing caused by sequence repeats (Matzke & Matzke, 1995; Yevtushenko & Misra, 2007). These are important considerations for any potential releases of transgenic lines for the purposes of species restoration (Newhouse, Polin‐McGuigan, et al., 2014). Eleven low‐copy number lines (i.e., 1–2 copies) were identified and their plantlets were regenerated in tissue culture and preliminarily screened for differential expression in response to wounding with reverse transcription (RT)‐qPCR (Table S1). Lines with the lowest expression were excluded; three single‐copy and two double‐copy candidate lines were selected for evaluation of transcriptional response to infection (Table 1). The *win3.12‐OxO* double‐copy events WX162 and WX167 showed highest differential expression (Table 1) and were regenerated for use in bioassays, rooted ex vitro (Oakes et al., 2016), potted, and grown in greenhouse conditions.

**TABLE 1:** *OxO* mRNA expression levels of blight‐infected *win3.12‐OxO Castanea dentata* lines relative to CaMV35S‐*OxO* event SX215 (Zhang et al., 2013) at 4 days postinoculation

*win3.12‐OxO* line	Transgene copy number	Untreated relative expression	Blight infection relative expression	Fold change
SX215 (CaMV35S*‐OxO* control)	1	1	—	—
WX17	1	0.00656	0.01663	2.53
WX31	1	—	0.00002	—
WX144	1	0.00012	0.00040	3.18
WX162	2	0.03200	2.17471	67.96
WX167	2	0.03443	0.24048	6.98

### 2.2 Transcriptional response of transgenic American chestnut *win3.12‐OxO* events to chestnut blight infection and oxalic acid treatment in tissue culture

**FIGURE 2:**
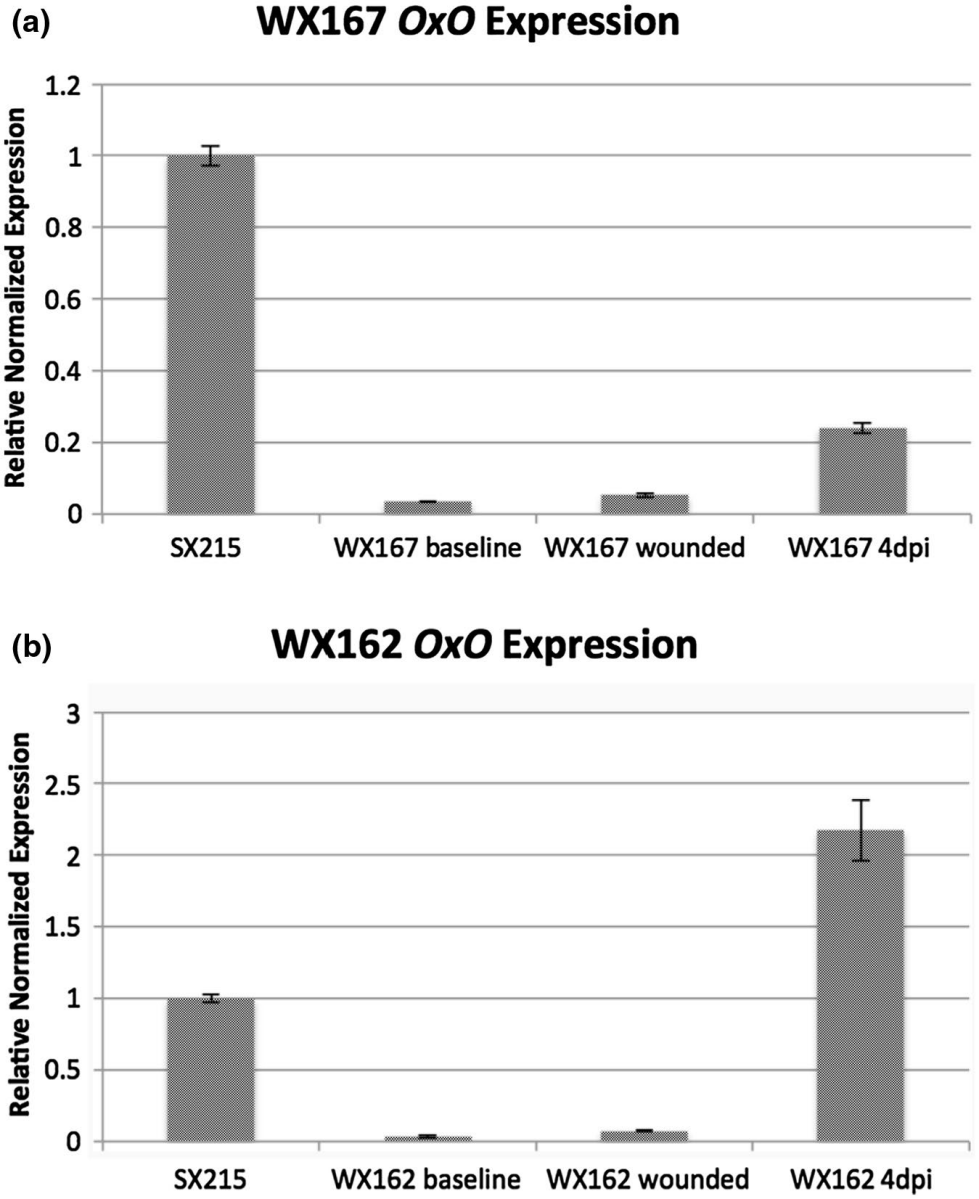
Transcriptional response of *win3.12‐OxO* in tissue culture plantlets of transgenic lines WX167 (a) and WX162 (b) to infection with chestnut blight. RNA samples were collected from untreated (baseline), wounded, and EP155 *Cryphonectria parasitica*‐infected stems 4 days postinoculation (dpi). CaMV35S*‐OxO* line SX215 was used as a standard for the relative *OxO* expression level that typically enhances blight tolerance to similar levels as Chinese chestnut (Zhang et al., 2013). Expression levels were quantified with reverse transcription quantitative PCR; values represent average *OxO* transcript expression of three plantlets from each transgenic line run in technical triplicate, error bars indicate one standard error of the mean. *Actin* and *EF1* were used as reference genes for normalization of gene expression data

Tissue culture plantlets of five candidate transgenic lines were grown to maximum height in tissue culture vessels and were inoculated with chestnut blight fungus (Figure S2). Infections were allowed to take place over 4 days prior to RNA extraction and RT‐qPCR. All transgenic lines showed *win3.12‐OxO* transcript induction surpassing baseline and wounded controls (Table 1). The transgenic line WX162 showed the highest levels of induction (Figure 2), with an accumulation of transcript resulting in a c.68‐fold increase in expression. The blight‐induced level of expression in WX162 surpasses many of the previously characterized CaMV35S*‐OxO* events (Zhang et al., 2013) with elevated levels of blight tolerance. To determine if oxalic acid played a role in the induction response, tissue culture plantlets of WX162 and WX167 were treated with 2 mM oxalic acid solution. Oxalic acid‐treated stems of both lines showed increased expression compared to controls, but expression levels were lower than pathogen‐induced treatments (Figure S1).

### 2.3 Histochemical staining assay for oxalate oxidase activity

Oxalate oxidase enzyme activity was detected in tissue culture plantlets and leaves from *win3.12‐OxO* lines with histochemical staining. Activity was detectable in all lines tested except WX31 (Table 2), which also had low levels of *OxO* mRNA expression (Table 1). Intensity of staining increases with higher OxO enzymatic activity: the darkest stains were observed on transgenic lines WX162 and WX167 (Table 3), which correlates with the mRNA expression levels observed in RT‐qPCR (Table 1). Staining of *win3.12‐OxO* transgenic lines took place over a period of several hours; in contrast, the CaMV35S*‐OxO* control with constitutive expression stained within an hour of being placed in histochemical solution. This suggests an induction of enzymatic activity in *win3.12‐OxO* lines with low levels of OxO initially present. Tissue culture plantlets and leaves from the offspring of WX162 (T_1_ first generation outcross) were treated with histochemical staining solution. Staining appeared consistent with the WX162 T_0_ pollen parent, indicating strong OxO activity present in the leaves and stems (Table 3). Nut cores taken from the kernels (cotyledons) of chestnuts produced through controlled crosses with WX162 pollen stained in histochemical solution, indicating that the promoter is active in nut tissue.

**TABLE 2:** Histochemical OxO activity staining of tissue culture leaves and stems of low‐expressing *win3.12‐OxO* lines

OxO event	+	−
SX215	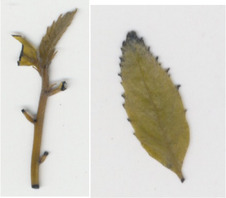	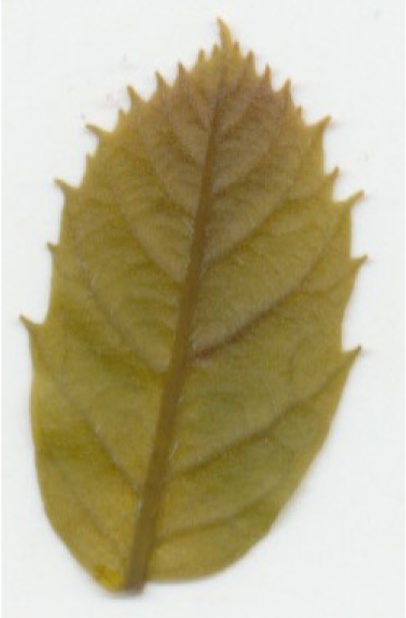
WX17	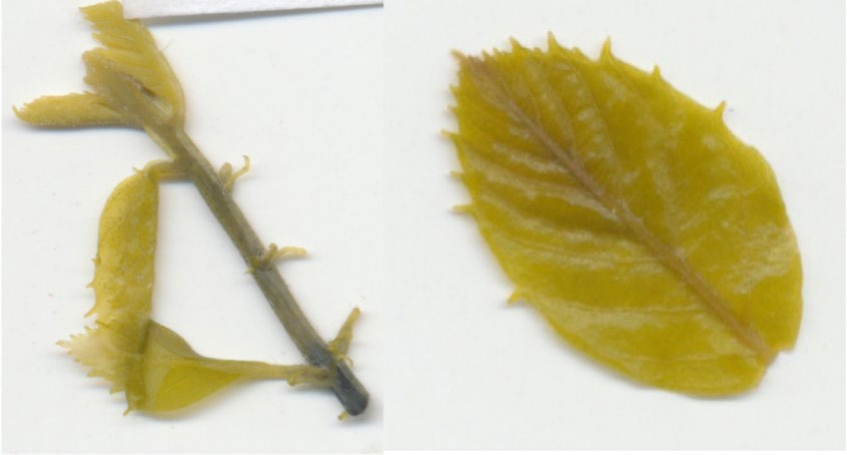	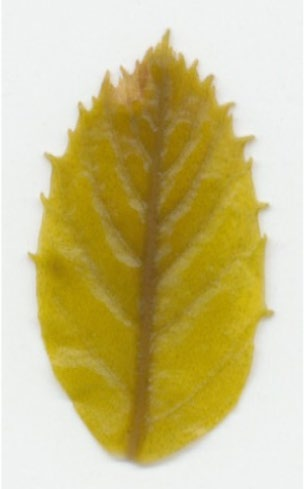
WX31	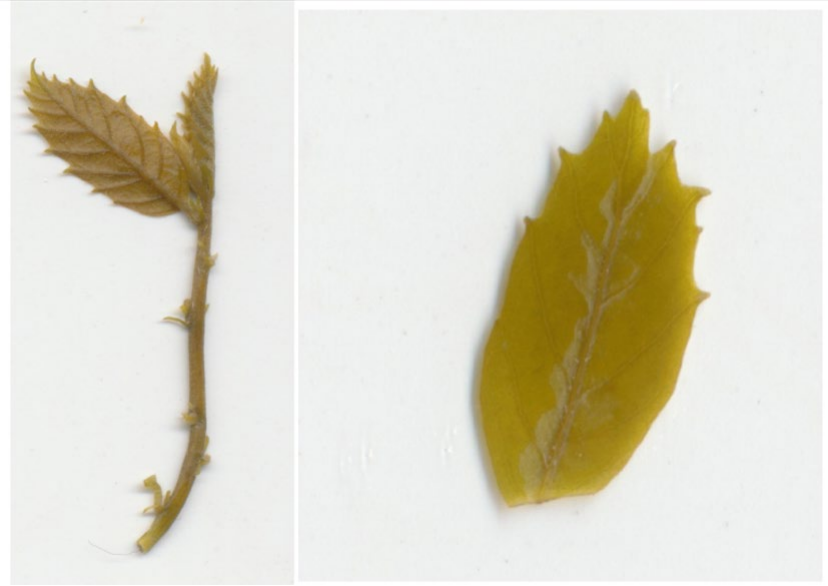	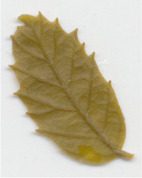
WX144	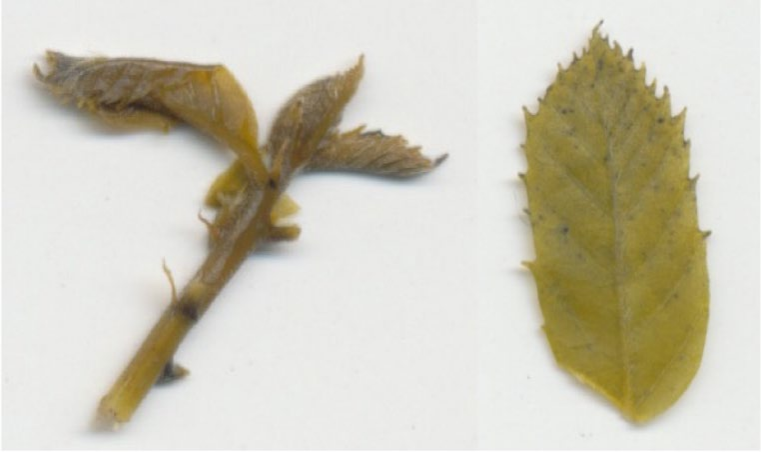	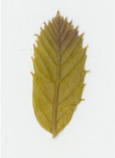

Note

Dark staining indicates OxO activity (+); negative control contains no oxalic acid (OA) to indicate unrelated H_2_O_2_ production.

**TABLE 3:** Histochemical OxO activity staining of tissue culture leaves and stems of high‐expressing *win3.12‐OxO* lines

OxO event	+	−
SX215	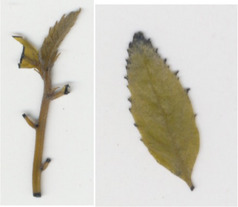	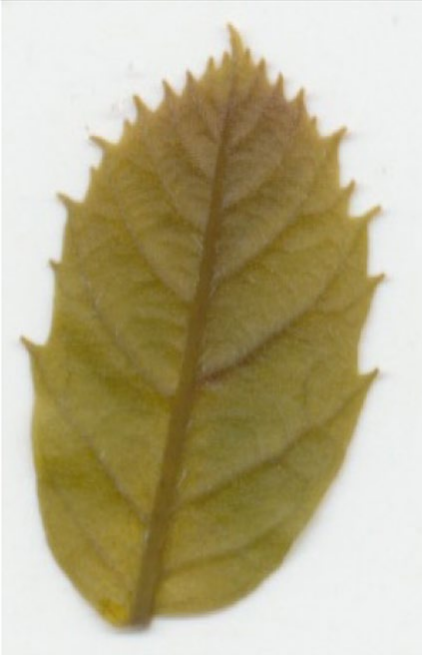
WX167	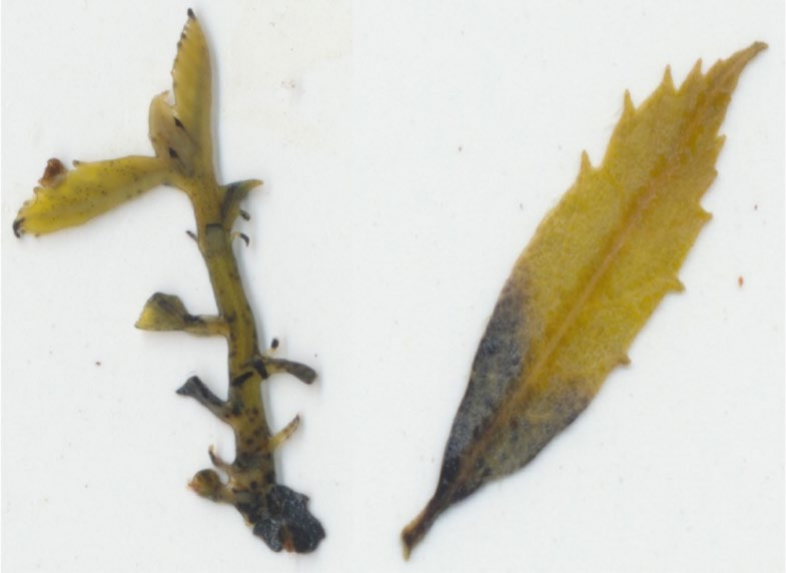	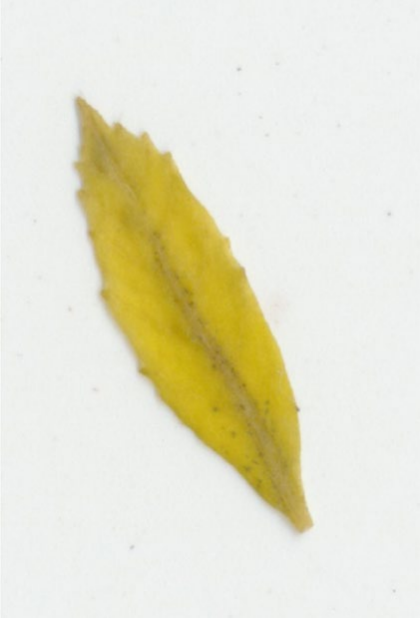
WX162 T_0_	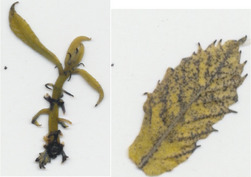	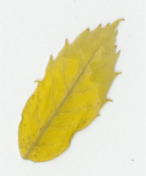
WX162 T_1_	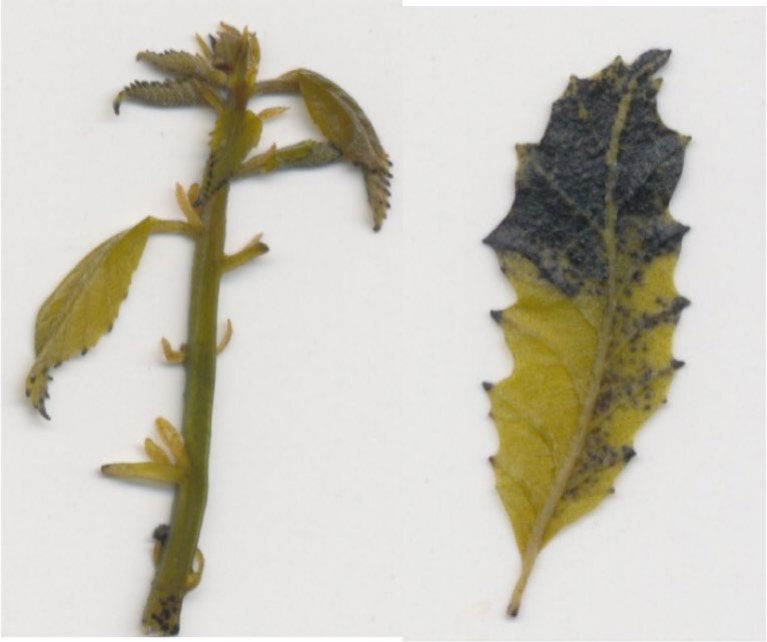	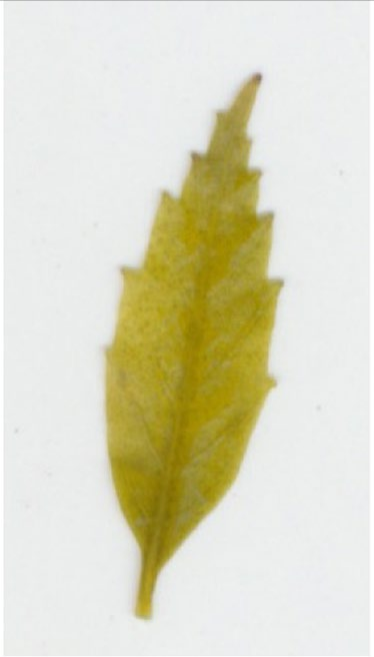

Note

Dark staining indicates OxO activity (+); negative control contains no oxalic acid (OA) to indicate unrelated H_2_O_2_ production.

### 2.4 Detached leaf blight fungus inoculation bioassays

**FIGURE 3:**
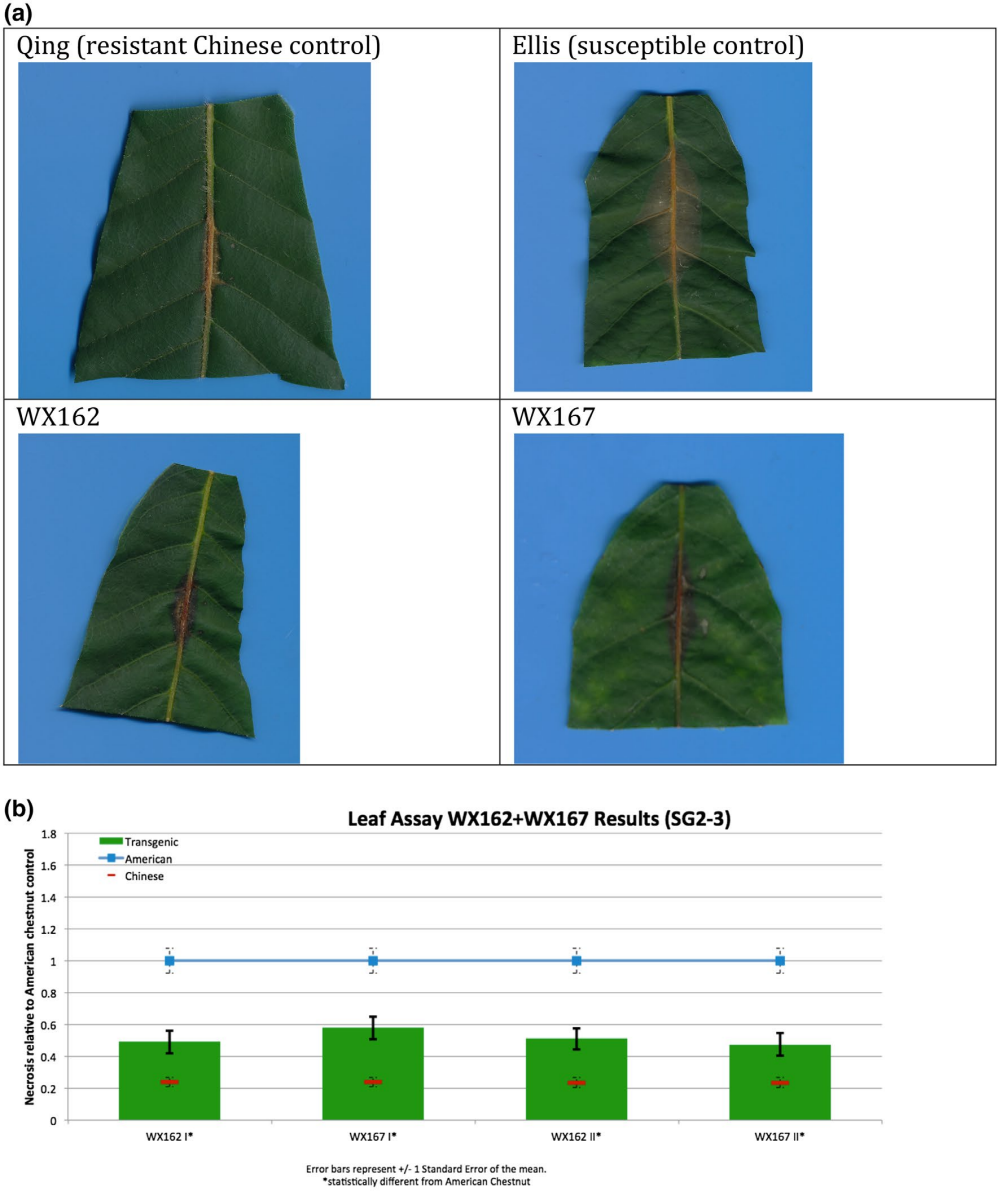
Detached leaf blight fungus inoculation assay results. (a) Leaf lesions outlined on representative samples of resistant and susceptible controls, *win3.12‐OxO* lines WX162 and WX167. (b) Statistical analysis of leaf canker area on replicated detached leaf fungal inoculation assays: lesion area comparison between transgenic WX162 (*n* = 12) and WX167 (*n* = 13) (green columns) and resistant controls (*n* = 11) (red dash), relative to normalized susceptible (*n* = 11) (blue line) controls in two replicate experiments

Transgenic *win3.12‐OxO* lines WX162 and WX167 were subjected to detached leaf inoculation bioassays (Figure 2; Newhouse, Spitzer, et al., 2014) to assess the effect of *OxO* expression on lesion size of the inoculated leaves from potted greenhouse plants. When lesion sizes were compared with the susceptible nontransgenic clonal line (Ellis), WX162 and WX167 had significantly smaller lesions. Leaf lesions on resistant Chinese chestnut controls were smaller than both transgenic and nontransgenic *C. dentata* (Figure 3). When rating levels of resistance to chestnut blight according to Newhouse, Spitzer, et al. (2014), this would qualify as intermediate resistance. Although resistance measurements did not match that of Chinese chestnut in leaf assays, the test did indicate an elevated level of blight tolerance compared to wild‐type *C. dentata*.

### 2.5 Small stem fungal inoculation assays

**FIGURE 4:**
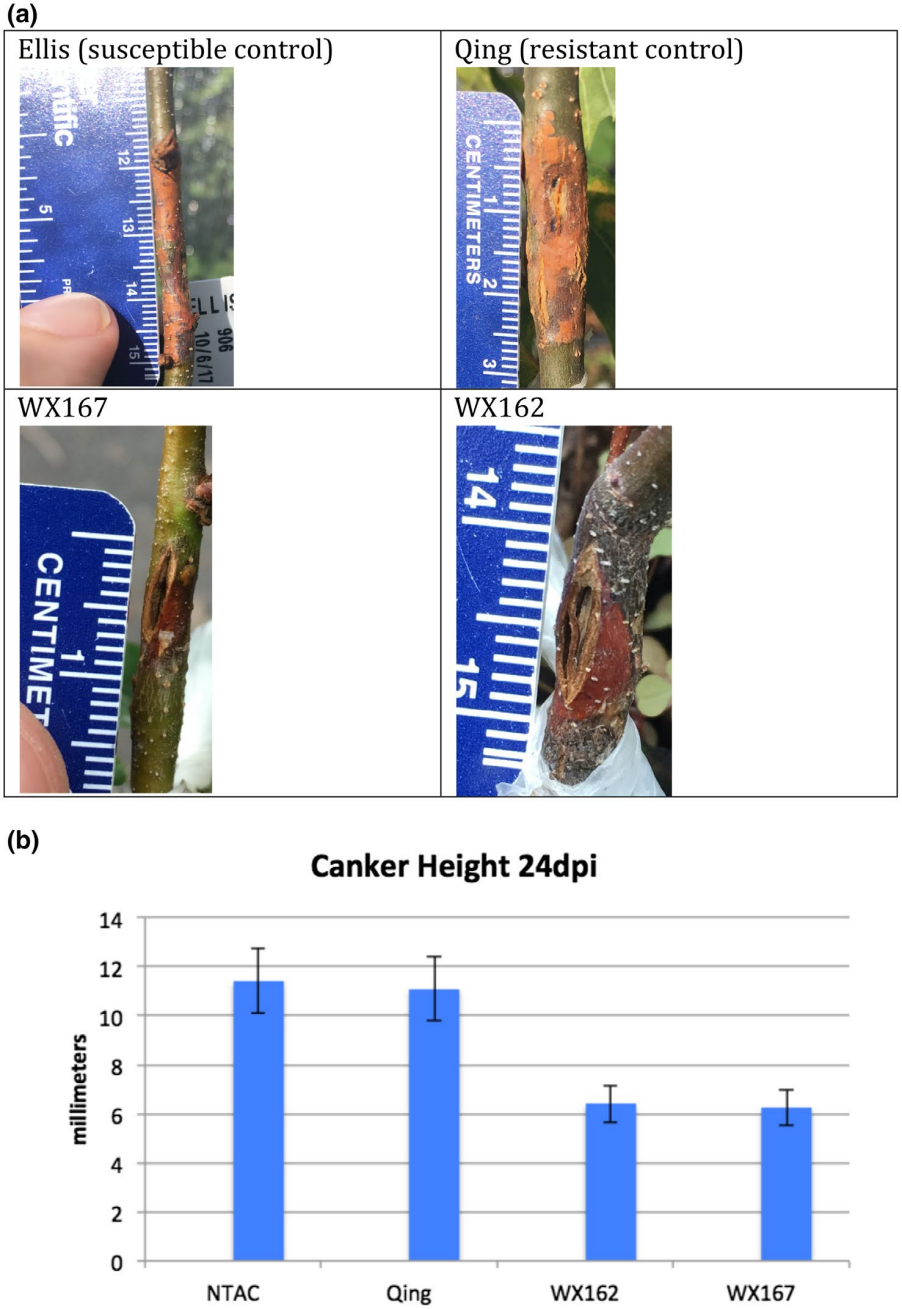
Small stem blight fungus inoculations. Stems of outdoor potted plants were wounded with a 5‐mm scalpel cut and fungal culture agar plugs of the highly virulent blight fungus strain EP155 were sealed into wounds with Parafilm. (a) Pictures taken 29 days postinoculation (dpi) show dramatic differences in canker size. Orange staining on stems expanding from wound sites indicate areas of infection. Widening of wounded tissue is more prominent in cv. Qing, WX167, and WX162 due to active growth, whereas growth has ceased on susceptible control. (b) Measurements taken 24 dpi show smaller canker height in *win3.12‐OxO* events compared to nontransgenic American chestnut (NTAC) and resistant Chinese chestnut Qing controls. Values are from a minimum of four plants from each transgenic line and controls, bars are one standard error of the mean

**FIGURE 5:**
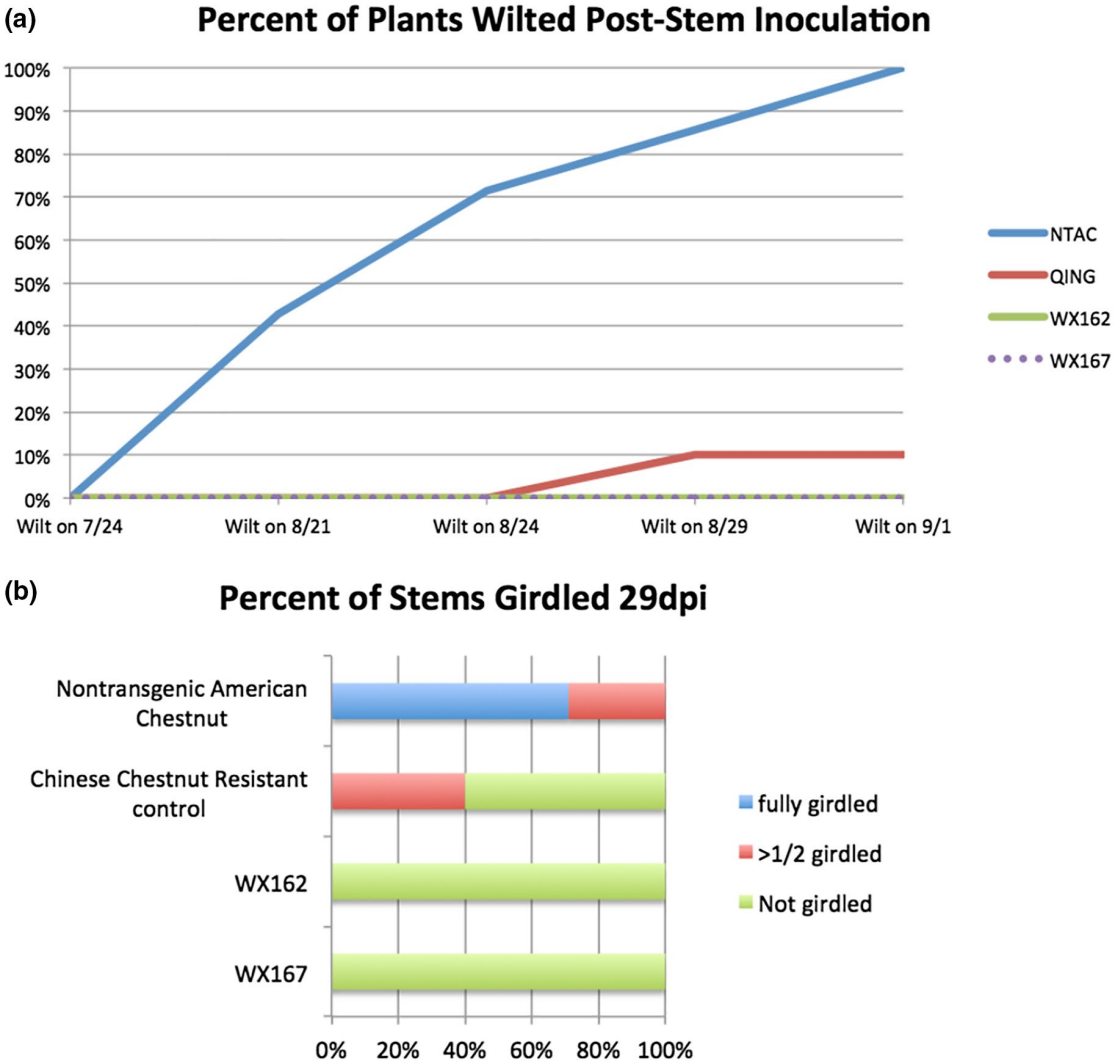
Percentage of stems girdled and percentage of plants wilted following small stem inoculations. Cankers, leaf wilt, and stem death were observed following inoculation. (a) Wilt over 5 weeks preceded death of all nontransgenic American chestnut (NTAC), while WX162 and WX167 showed no wilt. (b) Percentage of stems girdled 29 days postinoculation (dpi), full girdling leads to wilt and death. Qing, resistant Chinese chestnut control

In addition to detached leaf inoculation assays, small stem fungal inoculation assays (Powell et al., 2007, 2019) were performed on potted plants that had been generated from tissue culture. Detached leaf inoculation bioassays are a rapid indicator of increases in blight tolerance and can be performed when there is limited plant material available. Small stem inoculation assays further evaluate resistance when multiple plants are available for testing, and closer replicate the natural infection process that occurs on chestnut stems in the field. WX162 and WX167 along with susceptible and resistant controls were inoculated with chestnut blight fungus, in this case using the highly virulent EP155 strain of *C. parasitica*. Canker height, stem girdling, wilting, and death were monitored over 5 weeks until all susceptible control stems had died above cankers. During this period, WX162 and WX167 had significantly smaller cankers than both susceptible and resistant controls (Figure 4). No transgenic plants suffered wilt or died during the experimental period (Figure 5). These results appear to be consistent with elevated levels of blight tolerance observed in small stem assays of high‐expressing CaMV35S*‐OxO* lines (Powell et al., 2019).

## 3 DISCUSSION

This study evaluated the expression patterns of the *win3.12* promoter in transgenic American chestnut and its effectiveness in driving a blight tolerance transgene. Single‐copy *win3.12‐OxO* transgenic lines had prohibitively low levels of expression, but double‐copy lines showed transcription at levels similar to a previously described transgenic *C. dentata* line that used a different promoter (Zhang et al., 2013). In gene expression studies, a strong induction of *OxO* in response to infection with chestnut blight was observed; one transgenic line, WX162, produced levels of mRNA expression comparable to blight‐tolerant CaMV35S*‐OxO* events (Zhang et al., 2013). In detached‐leaf blight fungus inoculation assays, a significant decrease in lesion size compared to nontransgenic controls was observed, although lesions were still slightly larger than resistant Chinese chestnut (*C. mollissima*) controls. The results of small stem inoculations were similar to results of tests performed on previously characterized CaMV35S‐*OxO* lines (Powell et al., 2019), which indicated elevated levels of blight tolerance. The canker heights of resistant *C. mollissima* controls were similar to the susceptible controls in whole‐plant small stem inoculations (Figure 4b), but these cankers did not girdle the stems or wilt most of the resistant control plants (Figure 5). Despite the lack of significant difference in canker heights between resistant and susceptible controls, the canker heights of transgenic lines were significantly smaller than both controls. In addition to reduced canker size, inoculated transgenic plants did not wilt from infections because cankers did not girdle their stems. Cankers that result in the girdling of stems disrupt the flow of water and nutrients to tissues distal to the infection site. When girdling occurs on the main stem of a tree, it kills the apical stem, resulting in a loss of the tallest portions of the tree, including the canopy. As a forest tree species, the ability for *C. dentata* to compete for canopy is essential. By introducing a construct that prevents stem girdling from chestnut blight, it may be possible to restore the ability of *C. dentata* to compete for canopy. Small stem inoculation assays indicated greater increases in blight tolerance in transgenic lines compared to tests conducted with leaf assays; this may be due to higher levels of transgene expression in stem tissue than leaf tissue, which was previously observed in transgenic tobacco using the *win3.12* promoter (Yevtushenko et al., 2004). Because most naturally occurring blight infections cause canker damage on stem tissues and not leaves, this expression pattern may be useful for regulating blight defence transgenes. The formation of a wound periderm and lignified zone are essential steps in countering blight in resistant chestnut species by compartmentalizing infections (Hebard et al., 1984). In susceptible chestnuts, *C. parasitica* is capable of breaching the host wound periderm prior to the formation of a lignified zone, resulting in further invasion of stem tissue by mycelia (Hebard et al., 1984). Using a wound‐inducible promoter may target the expression of *OxO* to the wound periderm and facilitate the formation of a lignified zone, bolstering the tree's natural defences.

To test the enzymatic activity of the *OxO* gene product, histochemical assays that stain plant tissue with oxalate oxidase activity were performed. The darkness of staining in these assays correlated with the expression levels of transgenic lines observed in RT‐qPCR, with transgenic lines showing darker staining with increasing *OxO* transcript levels. RT‐qPCR and histochemical assays both indicated that the production of OxO enzyme is responsive to exposure to the oxalic acid. Transgenic *win3.12‐OxO* lines slowly stained over several hours as enzyme levels and activity increased in response to the oxalic acid in the solution whereas the CaMV35S*‐OxO* control SX215 stained within the first hour in histochemical solution. The constitutive expression of *OxO* in SX215 plantlets resulted in the enzyme being present prior to the initiation of treatment, leading to rapid staining. When plantlets from the first WX162 outcross offspring (T_1_) were tested with histochemical solution, the dark staining of the leaves and stems appeared consistent with the staining of the WX162 (T_0_) male parent, preliminarily indicating the stable heritability of the trait. Cores taken from chestnuts produced from crosses with WX162 pollen stained in the histochemical solution, indicating the gene is expressed in the nut tissue. The expression of *win3.12‐OxO* in nut tissue will aid in identifying nuts that received the transgene from their pollen parent prior to germination. The accumulation of gene product in nuts is also important to consider if this promoter is to be used with other transgenes.

Using an inducible promoter to drive a blight tolerance gene in *C. dentata*, a long‐lived tree species, may present multiple benefits over using a strong constitutive transgene promoter, such as a reduction in metabolic cost and an increase in long‐term stability (Godard et al., 2007; Zeller et al., 2013). In previous research, constitutive viral promoters in transgenic plants were observed to be prone to gene silencing due to a number of factors such as methylation and posttranscriptional mechanisms (Matzke & Matzke, 1995; Puddephat et al., 1996). Gene silencing could potentially impact the durability of disease resistance after decades of growth or after multiple generations of transgene inheritance (Metz et al., 1997; Weinhold et al., 2013). The effects of gene silencing have not been observed in transgenic American chestnut using the CaMV 35S promoter, but incorporating additional transgene expression systems such as inducible promoters will introduce redundancy into the *OxO* blight resistance strategy for increased resilience (Peremarti et al., 2010). As genomic sequencing of *Castanea* becomes more accessible, it may be possible to identify similar pathogen‐inducible genes and their promoters in the American chestnut itself for use in homologous expression systems.

The tests carried out in this study used plants that are clones of the original transgenic embryo lines (T_0_) produced through genetic transformation and tissue culture. Pollen from the WX162 line was recently used to produce controlled crosses with nontransgenic *C. dentata* mother trees and the resulting nuts will soon be germinated into seedlings after a stratification period. Seedlings are much more vigorous in growth than tissue culture‐propagated plants (Steiner et al., 2017) and produce trees of stature sooner than T_0_ tissue culture clones. The ability to grow vigorous trees from seedlings will facilitate the testing of growth rates, large stem inoculations, and long‐duration field experiments. Trees grown in the controlled research orchards of central New York will be exposed to natural outdoor conditions that closely resemble the blight pressure conditions in planting areas throughout *C. dentata*'s range.

In conclusion, the oxalate oxidase gene from wheat confers elevated chestnut blight resistance in American chestnut (Newhouse, Polin‐McGuigan, et al., 2014; Powell et al., 2019; Zhang et al., 2013). A redundant transgene expression strategy utilizing various types of tissue‐specific, constitutive, and inducible promoters could maximize the stability of blight tolerance in future generations of American chestnut by providing multiple sources of genetic resistance. In addition to using *win3.12* with *OxO*, the promoter may also be effective in driving expression of transgenes used in pyramided vectors for stacked blight resistance, for instance the use of *win3.12* with multiple pest and disease resistance genes targeted to combat threats such as *Phytophthora* root rot and chestnut gall wasp. This is the first report of inducible transgene expression used in a forest tree intended for species restoration. The *win3.12* promoter could be considered for use in other tree species, such as elm, ash, oak, hemlock, and others that are threatened by invasive pests and pathogens. Improvement of the promoter through duplication of the response elements could enable higher expression in single‐copy transgenic lines and is currently being investigated. Seedlings produced from crosses made with diverse wild‐type *C. dentata* mother trees and *win3.12‐OxO* transgenic lines will be closely monitored and evaluated for blight tolerance and vigour in long‐term field studies alongside previously characterized trees that use the CaMV 35S promoter. If blight resistance proves to be robust and stable in these trees, the use of *win3.12‐OxO* could play a valuable role in future restoration efforts of the American chestnut.

## 4 EXPERIMENTAL PROCEDURES

### 4.1 Vector construction

A vector (Figure 1) containing *win3.12* promoter was constructed starting from the previously used CaMV35S*‐OxO* construct containing *OxO* driven by CaMV 35S promoter with *ACT2* terminator (from *A. thaliana*), along with *NPTII* antibiotic selection gene (Zhang et al., 2013). The CaMV 35S promoter was removed via restriction digest and replaced with the *win3.12* promoter via Gibson assembly cloning (New England Biolabs). The new vector was designated pwin3.12‐OxO. The pwin3.12‐OxO plasmid's promoter and coding region were sequenced via Sanger sequencing (SUNY Upstate Molecular Analysis Core Facility, Syracuse, NY, USA). The pwin3.12‐OxO plasmid was transformed into *Agrobacterium tumefaciens* EHA105 using electroporation for future *C. dentata* embryo transformations.

### 4.2 Plant material

Somatic embryos from the wild‐type *C. dentata* ‘Ellis 1’ (Ellis) clonal line were used for *Agrobacterium‐*mediated transformation (McGuigan et al., 2020; Polin et al., 2006; Zhang et al., 2011). Somatic embryos were co‐cultivated with *Agrobacterium* containing the pwin3.12‐OxO vector and all transformed embryos were selected using paromomycin. Transgenic events were designated with the prefix WX (W = *win3.12* X = *OxO*). Shoots were regenerated from transformed somatic embryos (McGuigan et al., 2020) and maintained in multiplication medium until elongated for rooting (Oakes et al., 2016). Potted plantlets were generated from tissue‐cultured shoots using ex vitro rooting techniques and acclimatized in high‐humidity growth chambers (Oakes et al., 2016).

The WX162 transgenic line produced pollen from a spontaneous bloom of catkins on a potted plant in the greenhouse. A small amount of pollen was collected on glass slides and used immediately to pollinate a flowering wild‐type *C. dentata* in the orchard. The remaining pollen was collected on glass slides, stored at −80°C, and used in the subsequent season's pollinations.

### 4.3 Initial transgenic line molecular screening

Transgenic *C. dentata* lines with *win3.12‐OxO* were initially screened using PCR to confirm *OxO* insertion: gDNA was extracted from tissue culture plant leaves using a DNeasy plant mini kit (Qiagen). *OxO‐*positive events were tested using qPCR to determine the *OxO* gene insert copy number, using the Darling 4 transgenic line (Newhouse, Polin‐McGuigan, et al., 2014) as the copy number control. RT‐qPCR was used to determine baseline and wounded *OxO* mRNA expression levels. Tissue culture plantlets were mechanically wounded with a scalpel cut dissecting the stem vertically (Figure S3), and RNA was extracted from stems after 1 h using the cetyltrimethylammonium bromide (CTAB) method (Gambino et al., 2008). Expression levels were measured using the 48‐well MiniOpticon Real‐Time PCR System (Bio‐Rad) with data analysis using CFX Manager software (Bio‐Rad). The mRNA transcript levels were compared with the CaMV35S*‐OxO* event SX215, which was previously shown to have *OxO* expression levels capable of conferring blight tolerance similar to resistant Chinese chestnut controls (Zhang et al., 2013). WX events expressing *OxO* at the highest levels were selected and advanced through the tissue culture pipeline for bioassays.

### 4.4 Histochemical oxalate oxidase enzymatic staining assay

To validate the production of OxO and its enzymatic activity in *win3.12‐OxO* transgenic *C. dentata* lines, histochemical staining was performed on tissue culture plantlets. Leaves and shoot tips from tissue culture plantlets were cut and immediately placed into a solution containing oxalic acid and the staining agent 4‐chloro‐1‐napthol that stains hydrogen peroxide (a breakdown product of oxalic acid in an oxalate oxidase reaction), producing dark‐blue tissue staining (Tables 2 and 3) (Liang et al., 2001). A negative control solution without oxalic acid was used to rule out hydrogen peroxide production not associated with *OxO* activity. High‐resolution images were captured with a Canon Canoscan 5600F.

### 4.5 Detached leaf blight fungus inoculation assays

Transgenic *win3.12‐OxO* lines that showed the highest differential expression of *OxO* between wounded and unwounded plantlets in initial molecular screens were selected for bioassays using *C. parasitica* SG2‐3 (medium virulence strain). Leaf inoculations were administered according to previously described methods (Newhouse, Spitzer, et al., 2014; Zhang et al., 2013). Leaves from WX162 (*n* = 12) and WX167 (*n* = 13) along with a resistant Chinese chestnut control (*n* = 11) and a nontransgenic American chestnut control (*n* = 11) were collected from potted greenhouse plants. The leaves were uniformly wounded with a 0.5‐cm cut by scalpel and inoculated with a 3‐mm potato dextrose agar (PDA) plug of *C. parasitica* SG2‐3 fungal culture. Inoculated leaf lesions were imaged and measured using software that identifies pixels of discoloured leaf tissue in high‐resolution images (Assess v. 2.0; American Phytopathological Society). The lesion surface area was measured and normalized against resistant (Chinese chestnut) and susceptible (wild‐type American chestnut) controls.

### 4.6 Whole‐plant small stem inoculation assays

Stem inoculation assays were performed on potted plants grown in outdoor shade houses using the methods previously described by Powell et al. (2007). Cuts 0.5 cm long were made on the lower stems of plants using a scalpel to achieve uniform depth of cuts. Plugs of cultured *C. parasitica* EP155 (highly virulent strain) growing on PDA were applied to the wounds and sealed with Parafilm. Tissue culture clones of WX162 (*n* = 5) and WX167 (*n* = 4) were tested against nontransgenic Ellis isogenic clones as well as unrelated nontransgenic *C. dentata* seedlings as susceptible controls (*n* = 7). Clones of Chinese chestnut (*Ca. mollissima* ‘Qing’) (*n* = 5) were used for resistant controls. Plants were monitored for canker growth and wilting as *C. parasitica* infections progressed. Canker height was measured along with the extent of stem girdling. Leaf wilting was observed, which occurs when cankers have cut off xylem and phloem transportation through the plant. Partial wilting generally occurs 1–2 days before full wilt and death sets in.

### 4.7 Tissue culture gene expression analysis

To determine the response of *win3.12‐OxO* to infection with *C. parasitica*, a new type of inoculation procedure was developed. Transgenic plantlets were removed from tissue culture MK‐5 vessels, wounded with a scalpel, and inoculated with fungal cultures. Wounds in the stems were a uniform 0.5 cm. *C. parasitica* EP155 was grown on PDA for 3 days and agar plugs were taken from the leading edge of mycelial growth using a 0.5‐cm hollow punch. The upper third of the plug was sliced to obtain the upper layer of gel‐containing fungal mycelia. The disk of fungal agar was placed over the wound to initiate mycelial growth into the wound site and trigger an infection response from *win3.12‐OxO* (Figure S2). All work was done aseptically, with the exception of the inoculation cultures. Inoculated plantlets were placed back into tissue culture cubes and allowed to grow under normal tissue culture conditions (Figure 1). Wounded controls without fungal inoculation were made concurrently. Inoculated stems were collected after 4 days of fungal infection. RNA was extracted using a CTAB RNA extraction protocol (Gambino et al., 2008) and reverse transcribed using an iSCRIPT gDNA clear cDNA kit (Bio‐Rad). Transcript expression levels were measured using RT‐qPCR; iTaq SYBR green MasterMix (Bio‐Rad) was used for SYBR Green PCR. Fluorescence was measured using the Bio‐Rad CFX Real‐Time System (Table S2) and data were analysed using Bio‐Rad CFX Maestro software. Samples were analysed in technical triplicate and normalized with two internal reference genes: *Actin* and *EF1*. Ellis (nontransgenic isogenic line) was used as negative control for *OxO* expression (Table S1) and CaMV35S*‐OxO* line SX215 (Zhang et al., 2013) was used as a positive control and for relative expression.

## CONFLICT OF INTEREST

The authors declare no conflict of interest.

## AUTHOR CONTRIBUTIONS

K.B. cloned the pwin3.12‐OxO vector, L.M. carried out embryo transformations and regenerations, K.S. performed initial molecular screening of transgenic lines, T.C. assisted in tissue culture maintenance and experiments, E.C. designed and carried out experiments and wrote the manuscript, and W.P. is the Principal Investigator. All authors read and approved the final manuscript.

## Supporting information


**FIGURE S1** *OxO* RNA expression in response to oxalic acid (OA) treatment in WX162 (a) and WX167 (b) tissue culture stems over a 24‐h period compared to water control. Error bars indicate standard error of the mean of three treated stems

**FIGURE S2** Tissue culture stem blight fungus inoculations. Tissue culture stems are inoculated with agar discs containing *Cryphonectria parasitica* EP155 mycelia. Agar disc immediately following stem inoculation (a). Agar plug 4 days postinoculation (b) is still mostly clear of fungal growth. The mycelia in (b) have begun consuming the agar disc and are growing into the wound on the stem

**FIGURE S3** Tissue culture stem scalpel wounding. Tissue culture plantlets were dissected longitudinally with a scalpel to induce *OxO* expression prior to RNA extraction and reverse transcription quantitative PCR

**TABLE S1** Primers used for reverse transcription quantitative PCR in this study

**TABLE S2** Thermocycler programme used for reverse transcription quantitative PCR in this study

## Data Availability

The data that support the findings of this study are available from the corresponding author upon reasonable request.
