# Supplementary material for: Pathogen‐induced expression of a blight tolerance transgene in American chestnut
Source: Mol Plant Pathol. 2021 Nov 28;23(3):370–82. doi: 10.1111/mpp.13165 (PMC8828690; doi:10.1111/mpp.13165)
Supplement: Supplementary file 1 — FIGURE S1 OxO RNA expression in response to oxalic acid (OA) treatment in WX162 (a) and WX167 (b) tissue culture stems over a 24‐h period compared to water control. Error bars indicate standard error of the mean of three treated stems [file MPP-23-370-s001.doc]

**(a)**

**
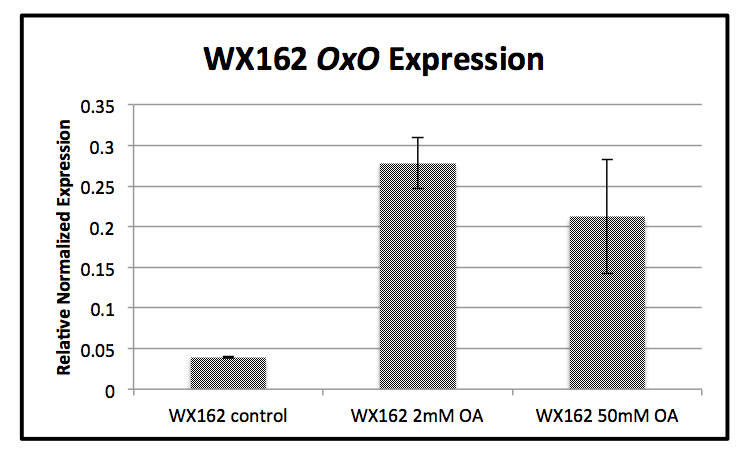
(b)
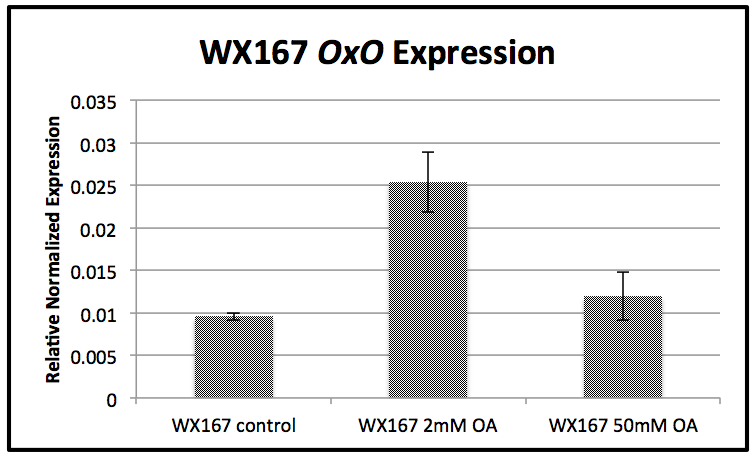
**

**Figure S1.** *OxO* RNA expression in response to oxalic acid (OA) treatment in WX162 (a) and WX167 (b) tissue culture stems over 24-hour period compared to water control. Error bars indicate standard error of the mean of three treated stems.
