# Supplementary material for: Pathogen‐induced expression of a blight tolerance transgene in American chestnut
Source: Mol Plant Pathol. 2021 Nov 28;23(3):370–82. doi: 10.1111/mpp.13165 (PMC8828690; doi:10.1111/mpp.13165)
Supplement: Supplementary file 2 — FIGURE S2 Tissue culture stem blight fungus inoculations. Tissue culture stems are inoculated with agar discs containing Cryphonectria parasitica EP155 mycelia. Agar disc immediately following stem inoculation (a). Agar plug 4 days postinoculation (b) is still mostly clear of fungal growth. The mycelia in (b) have begun consuming the agar disc and are growing into the wound on the stem [file MPP-23-370-s004.docx]

(a) (b)


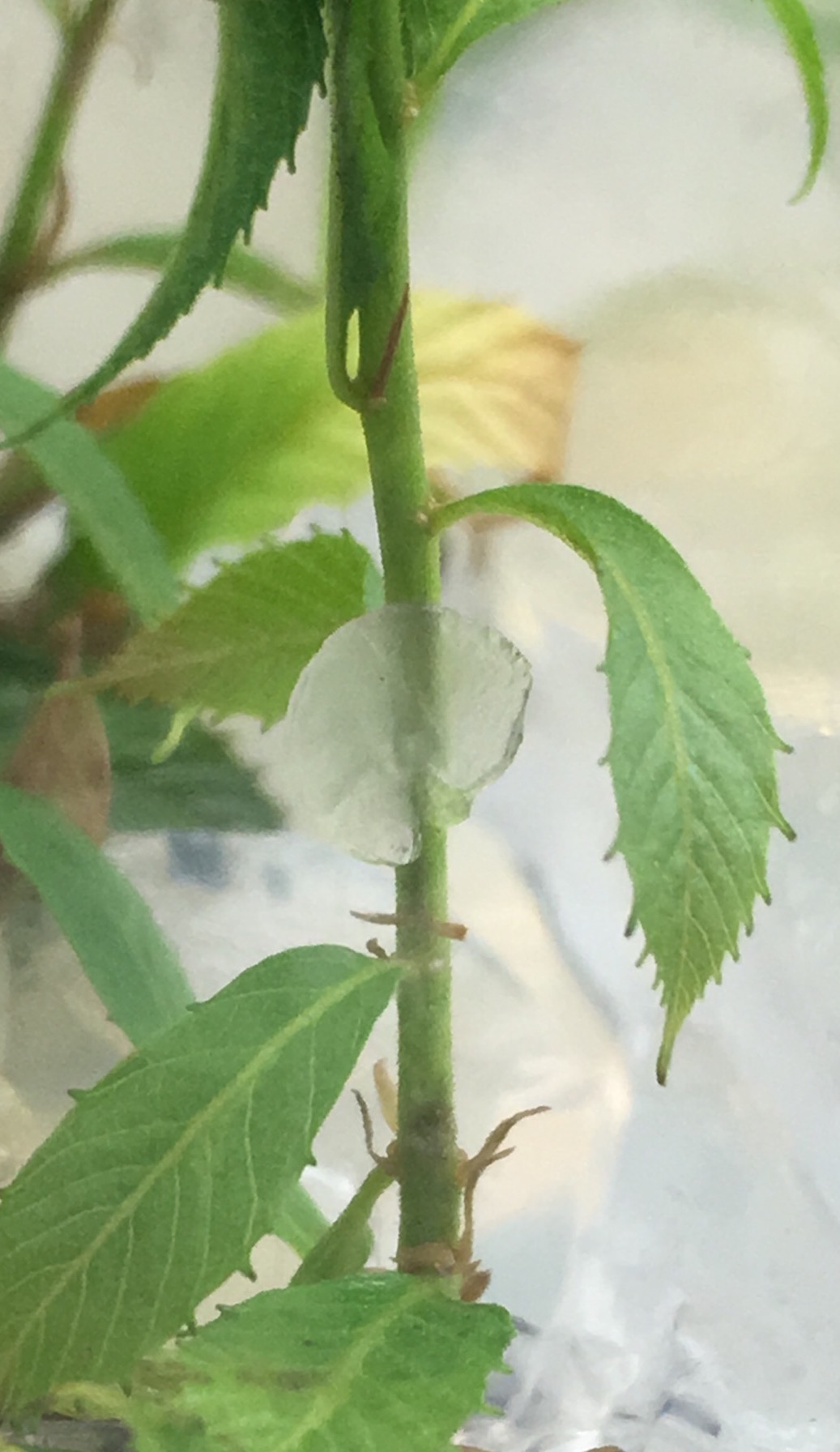

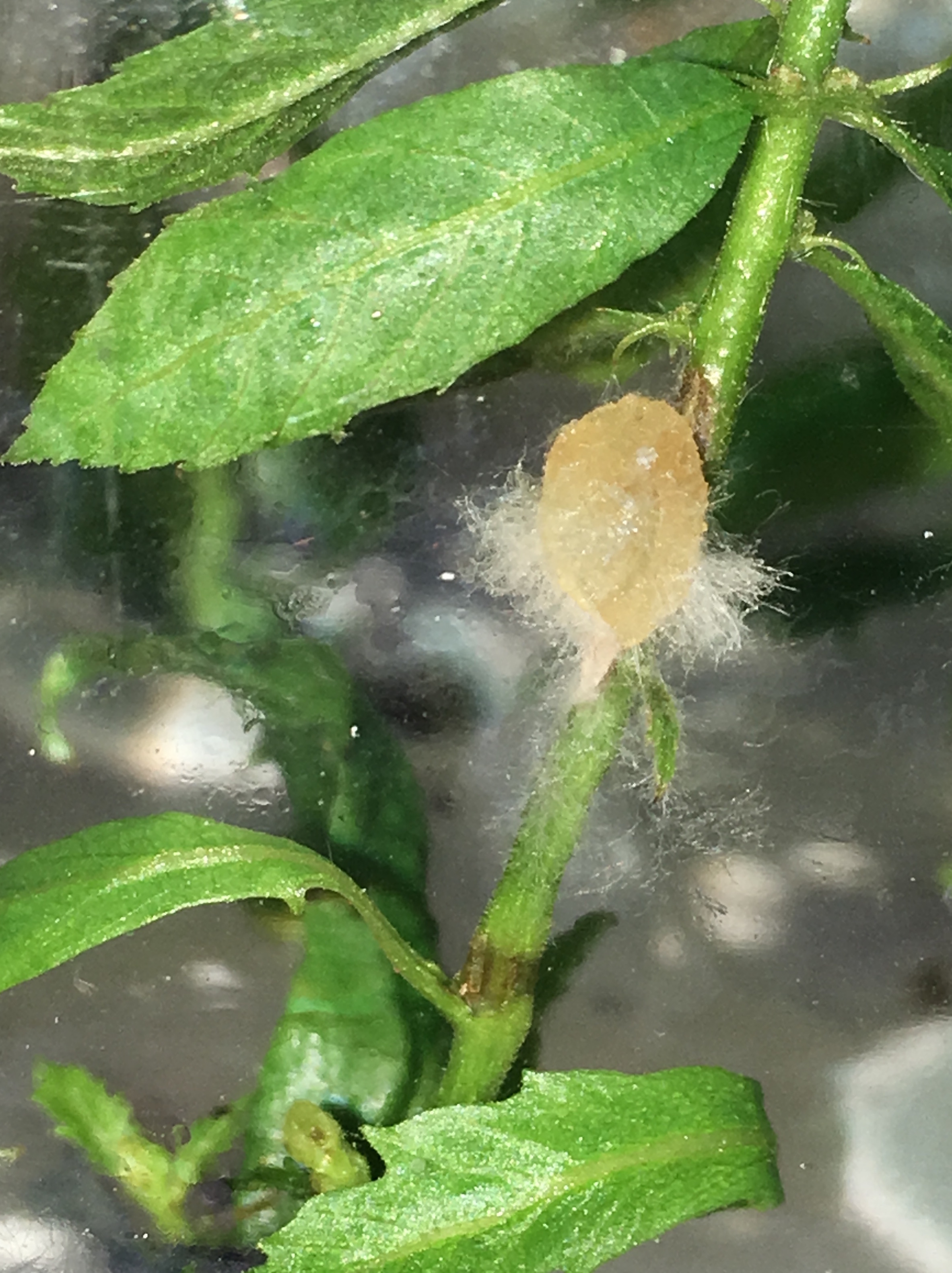


**Figure S2.** Tissue culture stem blight fungus inoculations. Tissue culture stems are inoculated with agar discs containing *C. parasitica* strain EP155 mycelia. Agar disc immediately following stem inoculation (a). Agar plug four days post inoculation (b) is still most clear of fungal growth. The mycelia in (b) have begun consuming the agar disc and are growing into the wound on the stem.
