# Supplementary material for: Pathogen‐induced expression of a blight tolerance transgene in American chestnut
Source: Mol Plant Pathol. 2021 Nov 28;23(3):370–82. doi: 10.1111/mpp.13165 (PMC8828690; doi:10.1111/mpp.13165)
Supplement: Supplementary file 3 — FIGURE S3 Tissue culture stem scalpel wounding. Tissue culture plantlets were dissected longitudinally with a scalpel to induce OxO expression prior to RNA extraction and reverse transcription quantitative PCR [file MPP-23-370-s005.docx]

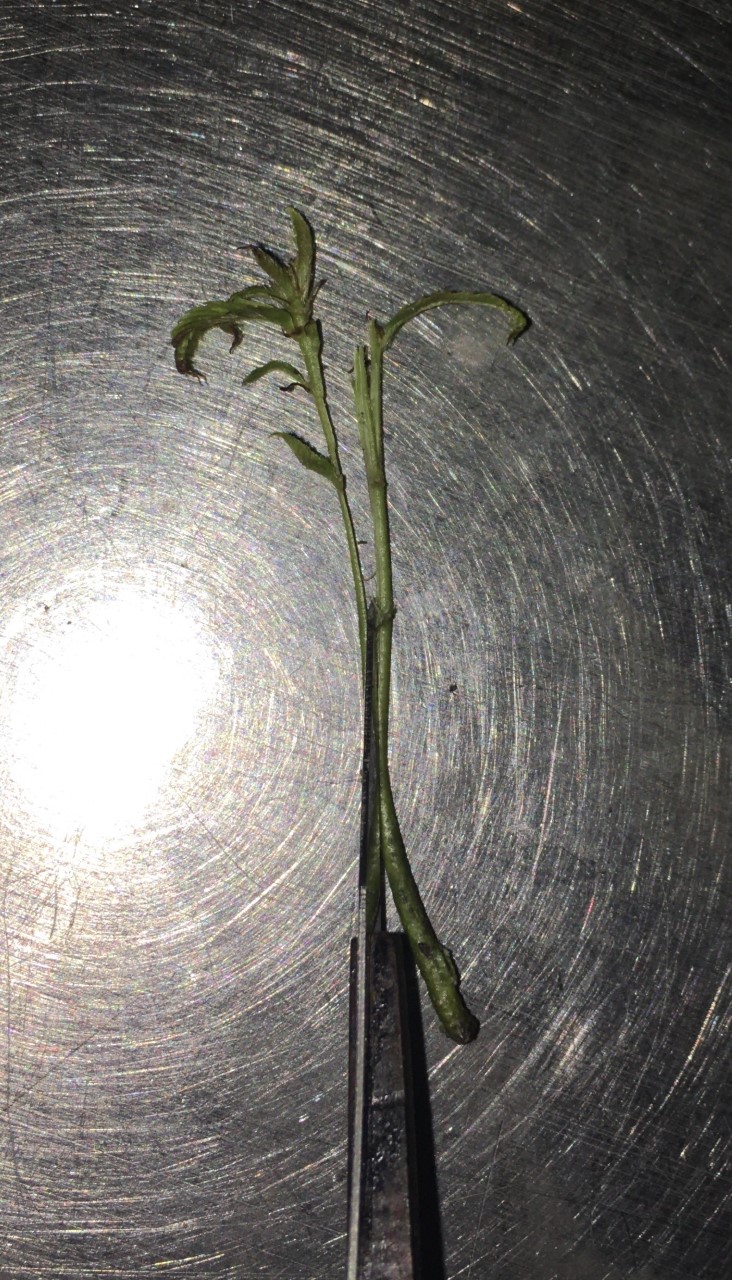


**Figure S3.** Tissue culture stem scalpel wounding. Tissue culture plantlets were dissected longitudinally with a scalpel to induce *OxO* expression prior to RNA extraction and RT-qPCR.
