# Supplementary material for: Pathogen‐induced expression of a blight tolerance transgene in American chestnut
Source: Mol Plant Pathol. 2021 Nov 28;23(3):370–82. doi: 10.1111/mpp.13165 (PMC8828690; doi:10.1111/mpp.13165)
Supplement: Supplementary file 4 — TABLE S1 Primers used for reverse transcription quantitative PCR in this study [file MPP-23-370-s003.docx]

**Table S1.** Primers used for RT-qPCR in this study.

| Primer name | Primer sequence |
| --- | --- |
| OxO Forward  OxO Reverse | GCCAACTTGGACGAGAAGAG  CCTAGTAGCTGGCCTGTTCG |
| Actin Forward  Actin Reverse | CCTTGCTGGTCGTGATCTC  GTCTCAAGTTCCTGGCTCATAGTC |
| EF1 Forward  EF1 Reverse | CGGTTACTGAGTACTAGCCTTG  CTGCCGAAGACCTTATTGAAAG |
