# Supplementary material for: Pathogen‐induced expression of a blight tolerance transgene in American chestnut
Source: Mol Plant Pathol. 2021 Nov 28;23(3):370–82. doi: 10.1111/mpp.13165 (PMC8828690; doi:10.1111/mpp.13165)
Supplement: Supplementary file 5 — TABLE S2 Thermocycler programme used for reverse transcription quantitative PCR in this study [file MPP-23-370-s002.docx]

**Table S2.** Thermocycler program used for RT-qPCR in this study

| Step | Time |
| --- | --- |
| 1. 95˚C | 3:00 |
| 2. 95˚C | 0:10 |
| 3. 60˚C + Plate Read | 0:30 |
| 4. Go to Step 2 | 39 times |
| 5. 95˚C | 0:10 |
| 6. Melt Curve 65-95˚C + Plate Read  End | 0.5˚C/0:05 |
